# Beyond Antioxidant Activity: Redox Properties of Catechins May Affect Changes in the DNA Methylation Profile—The Example of *SRXN1* Gene

**DOI:** 10.3390/antiox12030754

**Published:** 2023-03-20

**Authors:** Patrycja Jakubek, Jovana Rajić, Monika Kuczyńska, Klaudia Suliborska, Mateusz Heldt, Karol Dziedziul, Melita Vidaković, Jacek Namieśnik, Agnieszka Bartoszek

**Affiliations:** 1 Faculty of Chemistry, Gdańsk University of Technology, 80-233 Gdańsk, Poland; 2 Laboratory of Mitochondrial Biology and Metabolism, Nencki Institute of Experimental Biology of Polish Academy of Sciences, 02-093 Warsaw, Poland; 3 Institute for Biological Research “Siniša Stanković”, National Institute of Republic of Serbia, University of Belgrade, 11060 Belgrade, Serbia; 4 Faculty of Applied Physics and Mathematics, Gdańsk University of Technology, 80-233 Gdańsk, Poland

**Keywords:** catechins, DNA methylation, electrochemistry, epigenetics, sulfiredoxin 1, reductive stress

## Abstract

The role of catechins in the epigenetic regulation of gene expression has been widely studied; however, if and how this phenomenon relates to the redox properties of these polyphenols remains unknown. Our earlier study demonstrated that exposure of the human colon adenocarcinoma HT29 cell line to these antioxidants affects the expression of redox-related genes. In particular, treatment with (−)-epigallocatechin (EGC) downregulated transcription of gene encoding sulfiredoxin-1 (SRXN1), the peroxidase involved in the protection of cells against hydrogen peroxide-induced oxidative stress. The aim of this study was to investigate whether the observed *SRXN1* downregulation was accompanied by changes in the DNA methylation level of its promoter and, if so, whether it was correlated with the redox properties of catechins. The impact on DNA methylation profile in HT29 cells treated with different concentrations of five catechins, varying in chemical structures and standard reduction potentials as well as susceptibility to oxidation, was monitored by a methylation-sensitive high-resolution melting technique employing the *SRXN1* promoter region as a model target. We demonstrated that catechins, indeed, are able to modulate DNA methylation of the SRXN1 gene in a redox-related manner. The nonlinear method in the statistical analysis made it possible to fish out two parameters (charge transfer in oxidation process *Q_ox_* and time of electron transfer *t*), whose strong interactions correlated with observed modulation of DNA methylation by catechins. Based on these findings, we present a proof-of-concept that DNA methylation, which limits *SRXN1* expression and thus restricts the multidirectional antioxidant action of SRXN1, may represent a mechanism protecting cells against reductive stress caused by particularly fast-reacting reductants such as EGC and (−)-epicatechin gallate (ECG) in our study.

## 1. Introduction

Plant-borne foods and beverages constitute a rich source of antioxidant phytochemicals such as polyphenols, whose consumption has been documented to bring beneficial effects for human health [1]. Dietary phenolic compounds belong to several classes, among which the most common are flavonoids. Electrochemical properties, hence the antioxidant activity of these compounds, are strictly related to their chemical structure. Flavonoids exhibiting particularly strong reducing potential are flavan-3-ols, commonly named—catechins. They constitute the major components of green tea, mainly represented by (−)-epigallocatechin gallate (EGCG), (−)-epigallocatechin (EGC), (−)-epicatechin gallate (ECG), (−)-epicatechin (EC), and (+)-catechin (C). Catechins are also abundant in fruits such as berries, apples, grapes, and grape seeds, as well as in cocoa and cocoa-based foodstuffs [2].

Catechins, especially those derived from green tea, have been frequently reported to exhibit a variety of chemopreventive activities both *in vitro* and *in vivo* [3]. These protective effects are believed to play a role in the prophylaxis of such chronic conditions as cancer, cardiovascular and infectious diseases, liver anomalies, as well as in diabetes [4,5,6,7]. The benefits seem associated mainly with the chemical neutralization of prooxidants, thus with the antioxidant potential of catechins, but may occasionally result from more specific activities, such as the ability to affect metabolic and signaling pathways [8]. The redox-related activities of these flavonoids embrace scavenging of reactive oxygen species (ROS), chelation of transition metal ions, inhibition of transcription factors induced under oxidative stress (e.g., activator protein 1—AP-1, and nuclear factor kappa-light-chain-enhancer of activated B cells—NF-κB), and other. The impact on the expression of redox-related genes has been recently shown as well [9].

Catechins have also been demonstrated to influence epigenetic mechanisms by the modulation of DNA methylation and histone acetylation levels [10,11,12,13]. However, how these impacts relate to the redox properties of catechins has not been studied to date, though the dependence of methyl transfer reactions on the redox status of cells is known [14]. DNA methylation is a reversible epigenetic modification often impaired in diseased states, e.g., in cancer tumor suppressor genes are frequently silenced by hypermethylation. Therefore, the inhibition of DNA methylation of such protective genes has become a promising target of anticarcinogenic prophylaxis [15]. By definition, the preventive strategy is mainly addressed to healthy people with increased risk of illness; thus, food ingredients are suggested to be the most accessible and safe protective factors. Among catechins, EGCG has been shown to be the strongest inhibitor of maintenance DNA methylation since its gallic acid moiety accommodates in the active site of DNA methyltransferase 1 (DNMT1), where it blocks DNMT1 catalytic activity and thereby decreases DNA methylation levels [13]. The inhibition of DNA methylation by catechins has been reported to also be driven indirectly as a consequence of methylation of catechins by catechol-*O*-methyl transferase (COMT), particularly in the case of EC and C. COMT competes with DNA methyltransferases (DNMTs) for the donor of a methyl group, S-adenosylmethionine (SAM), thereby diminishing their enzymatic activity [16]. Moreover, the demethylation of SAM leads to the formation of S-adenosylhomocysteine (SAH), which is an efficient and selective inhibitor of DNMTs [17].

DNA methylation affects both genome stability and gene expression; thus, any abnormalities concerning methylomes, such as hypo- or hypermethylation, may modulate mRNA levels and further influence disease development [18]. The impact of nutrition on gene expression has also been well established [19]. As shown for catechins in our previous study [9], exposure of the HT29 cell line to their different concentrations induced changes in the expression of redox-related genes. The physiological concentration (1 μM) of catechins upregulated several of them, while the higher 10 μM concentration seemed to sufficiently preserve cellular redox homeostasis, so the expression of redox-related genes remained not affected. The puzzling exception was the downregulation of the sulfiredoxin 1 (SRXN1) gene, encoding one of the members of the cellular thiolstat [20]. The main function of SRXN1 is to protect cells against hydrogen peroxide-induced oxidative stress by peroxiredoxin (I–IV) reactivation [21]. The activity state of peroxiredoxins is dependent on the oxidation state of sulfur in the peroxidatic cysteine (C_p_), located in the catalytic center of the enzyme. Hyperoxidation of C_p_ to sulfinic acid results in the inactivation of peroxiredoxin, which can be reversed in an ATP-dependent manner solely by SRXN1 with the aid of its single conserved cysteine residue present in the catalytic center [22]. Our observations suggested that the treatment with strong antioxidants by diminishing the expression of *SRNX1* sort of excluded this enzyme from the endogenous cellular antioxidant barrier. One may presume that this could represent a mechanism preventing organisms from being pushed into reductive stress when exposed to an excess of compounds whose electrochemical properties make them particularly effective reducing agents. The role of DNA methylation in the control of redox homeostasis in cells exposed to exogenous strong antioxidants was a tempting explanation in view of our earlier findings [9].

The objective of this study was to examine whether the previously observed [9] downregulation of the SRXN1 gene resulted from changes in the profile of the DNA methylation of CpG islands within its promoter. The cellular model was the same as in the previous study, i.e., undifferentiated human colon adenocarcinoma HT29 cell line exposed to redox-active dietary phytochemicals. Compounds selected for these investigations included catechin derivatives with different chemical structures and values of standard reduction potentials. Additionally, important but relatively weak compared to polyphenols [9,23], thiol antioxidant—glutathione—produced endogenously but also found in foods (exogenous source) [24] was chosen as a reference reducing agent, which, according to our previous studies, has no impact on *SRXN1* expression. We also wanted to determine whether changes in DNA methylation levels in the *SRXN1* promoter region are dependent on catechin structure or corresponding electrochemical properties. Our investigations provide new data related to the impact of catechins on DNA methylation in the context of their chemical structures, electrochemical properties, and concentration applied to cells. Based on the study results, we suggest the indirect role of DNA methylation in fine-tuning cellular redox homeostasis.

## 2. Materials and Methods

### 2.1. Selected Redox-Active Compounds

The study included the following redox-active compounds: (+)-catechin (C), (−)-epicatechin (EC), (−)-epigallocatechin (EGC), (−)-epicatechin gallate (ECG), (−)-epigallocatechin gallate (EGCG) from Extrasynthese (Genay Cedex, France) and glutathione (GSH) from Sigma-Aldrich (St. Louis, MO, USA). The DNA demethylating agent 5-aza-2′-cytidine (5-Aza) was purchased from Sigma-Aldrich (St. Louis, MO, USA).

### 2.2. Cell Culture

The human colon adenocarcinoma cell line (HT29) from the ATCC was cultured in McCoy’s medium supplemented with L-glutamine (2 mol/L), sodium bicarbonate (200 g/L), fetal bovine serum (100 mL/L) and antibiotics (100 U/mL penicillin and 100 g/L streptomycin) in a humidified atmosphere with 5% CO_2_ at 37 °C in a SMARTcell incubator (Heal Force). All reagents for cell culture were obtained from Sigma Aldrich (St. Louis, MO, USA).

### 2.3. Treatment of Cells with Selected Redox-Active Compounds

For gene expression analysis, HT29 cells were seeded in 24-well tissue culture plates (6 × 10^4^ cells per well in 1.8 mL of medium). Stock solutions of catechins and glutathione were prepared in analytical-grade ethanol (POCH, Gliwice, Poland) and ultrapure water purified with QPLUS185 system from Millipore (Burlington, MA, USA), then sterilized using Millex sterile R33 mm (0.22 µm) syringe-driven filters from Millipore (Burlington, MA, USA). After 24 h of settling down, the cells were treated with 0.2 mL of antioxidant solution and left for a further 24 h at 37 °C or for 72 h in the case of 5-Aza treatment (5-Aza treatment was repeated every day). The cells treated with catechins were exposed to 3% (*v*/*v*) of ethanol as a solvent, so the same concentration of ethanol was applied to control cells. In the case of glutathione, control cells were treated with water. A stock solution of 10 mM 5-Aza was prepared in dimethyl sulfoxide (DMSO, SERVA Electrophoresis GmbH, Heidelberg, Germany), and subsequent dilutions were prepared in culture medium. The final DMSO concentration to which cells were exposed did not exceed 0.1%.

For methylation analysis, HT29 cells were seeded in 6-well tissue culture plates (5 × 10^5^ cells per well in 3.6 mL of McCoy’s medium). After 24 h, cells were treated with 0.4 mL solutions of the investigated compounds in the 0.1–100 µM concentration range and incubated for 24 h at 37 °C. The subsequent steps were carried out in the same way as described earlier. All experiments were performed in three independent biological replicates.

### 2.4. Total RNA Isolation

Total RNA isolations from treated and control cells were carried out using RNeasy Mini Kit (Qiagen, Hilden, Germany). For homogenization, QIAshredder (Qiagen, Hilden, Germany) was used. To assure complete elimination of genomic DNA contamination, RNase-free DNase kit (Qiagen, Hilden, Germany) was applied. All steps were performed as stated in the manufacturer’s protocols. RNA quality and quantity was checked with Nanodrop 2000c (Thermo Scientific, Waltham, MA, USA) at absorbance ratios of 260/280 nm and 260/230 nm. Isolated RNAs were stored at −80 °C.

### 2.5. Microarray Analysis

Isolated mRNA (500 ng) was reverse transcribed to cDNA using an RT^2^ First Strand kit (Qiagen, Hilden, Germany). The obtained cDNA was subsequently mixed with an RT^2^ SYBR Green kit (Qiagen, Hilden, Germany) and pipetted into 96-well RT^2^ Profiler^TM^ PCR microarray human oxidative stress plates provided by Qiagen (Hilden, Germany). qPCR was performed with the aid of LightCycler^®^ 96 Instrument (Roche, Basel, Switzerland). All steps were carried out according to instructions provided by the manufacturer. The whole procedure was previously described in detail by Baranowska et al. [9].

### 2.6. Genomic DNA Isolation

Genomic DNA was isolated from treated and control cells using QuickDNA Miniprep Plus Kit from Zymo Research (Irvine, CA, USA) according to the manufacturer’s protocol. DNA quality and quantity were checked using Nanodrop 2000c (Thermo Scientific, Waltham, MA, USA) by measuring the ratio of absorbances at 260/280 nm and 260/230 nm. The DNA isolates were stored at −20 °C.

### 2.7. Bisulfite Conversion

Bisulfite conversion of isolated genomic DNA was performed with the EZ DNA Methylation kit (Zymo Research, Irvine, CA, USA) according to the protocol provided by the manufacturer. The reaction conditions were adjusted so as to obtain full cytosine (C) to thymine (T) conversion, as stated in the appendix of the instruction manual.

### 2.8. Prediction of CpG Islands and Primer Design for Methylation Analysis

Prediction of the CpG island of the human SRXN1 gene was performed with EMBOSS CpGplot as described before [25]. Primers for the SRXN1 gene were designed using MethPrimer. Two separate sets of primers were prepared: first corresponding to methylated (M) and second to unmethylated (U) promoter region. Their sequences are listed in Table S1 (Supplementary Materials). All the primers used for DNA methylation analysis were provided by Invitrogen (Waltham, MA, USA).

### 2.9. Methylation-Specific PCR (MSP)

MSP was performed using Maxima SYBR Green/ROX qPCR Master Mix (2x) from Thermo Scientific (Waltham, MA, USA). The reaction mix included Maxima SYBR Green/ROX qPCR Master Mix (2x), 10 µM of each primer, and 100 ng (M1/U1) or 200 ng (M3/U3) of bisulfited DNA. Thermal cycling conditions were set as described before [25]. A no-template control was used to detect any reagent contamination or formation of primer dimers. QuantStudio 3 Real-Time PCR System from Applied Biosystems (Waltham, MA, USA) was used to run MSP. The percentage of DNA methylation was calculated according to the formula: %M = 1/(2^dCt^ + 1), where dCt = Ct_M_ − Ct_U_. Ct_M_, cycle threshold value for MSP performed with methylated primers; Ct_U_, cycle threshold value for MSP performed with unmethylated primers.

### 2.10. Methylation-Sensitive High-Resolution Melting (MS-HRM)

Conditions and experiment design for MS-HRM have been described in detail previously [22]. Shortly, standard curves for MS-HRM analysis were prepared using human methylated and non-methylated DNA standards (Zymo Research, Irvine, CA, USA). Bisulfite-converted standards were mixed to obtain 0%, 50%, and 100% of DNA methylation. The reaction mixture consisted of 2x MeltDoctor^TM^ HRM Master Mix from Applied Biosystems (Waltham, MA, USA), 0.15 µM of each of the forward and reverse primers from the methylated and unmethylated set, and 10 ng (M1/U1) or 20 ng (M3/U3) of bisulfited DNA. PCR amplification and subsequent MS-HRM analysis were performed as described earlier [25]. Data normalization and quantitative calculation of DNA methylation percentage were carried out as described by Rajić et al. [26].

### 2.11. Quantitative Reverse Transcription PCR (RT-qPCR)

Reverse transcription was conducted using QuantiNova Reverse Transcription Kit (Qiagen, Hilden, Germany). The amount of mRNA used for cDNA synthesis was 500 ng. Synthesized cDNA was diluted 1:9 in DNase-free water prior to RT-qPCR according to the manufacturer’s recommendation. RT-qPCR was performed using FastStart Essential DNA Green Master (Roche, Basel, Switzerland) in LightCycler^®^ 96 Instrument (Roche, Basel, Switzerland). The thermal cycling conditions included initial denaturation at 95 °C for 15 min and subsequent 40 cycles of a three-step protocol: 95 °C for 10 s, 60 °C for 10 s, and 72 °C for 100 s. The three-step protocol was followed by melting: 95 °C for 10 s, 65 °C for 1 min, and 97 °C for 1 s. Primers for SRXN1 gene expression analysis were designed with the aid of Primer-BLAST based on the sequence available in GeneBank with the accession number NM_080725.3. Primers were provided by Genomed (Warsaw, Poland), and their sequences are listed in Table S2 (Supplementary Materials). Gene expression was calculated with the delta-delta Ct method after normalization to glyceraldehyde 3-phosphate dehydrogenase (*GAPDH*) used as a reference gene. The choice of reference gene was suited for the HT29 cell line based on the literature [27].

### 2.12. Statistical Analysis

The results are presented as the mean value ± SD of 3 independent biological replicates unless stated otherwise. The determination of the statistical significance between DNA methylation levels in control and in treated samples was evaluated by one-way ANOVA with Dunnett’s test using the Prism 6.0 software package (GraphPad Software, Inc., Boston, MA, USA). Differences were statistically significant at the level of *p* < 0.05. The search for strong interactions indicating differences between DNA methylation determinations and electrochemical as well as biological parameters established for the tested compounds in other studies was performed by *t*-test, Welsch, and Cochran tests with the aid of SAS Viya for Learners 3.5 analytics and data management platform

## 3. Results

### 3.1. The Impact of Catechins on SRXN1 Expression

**Figure 1:**
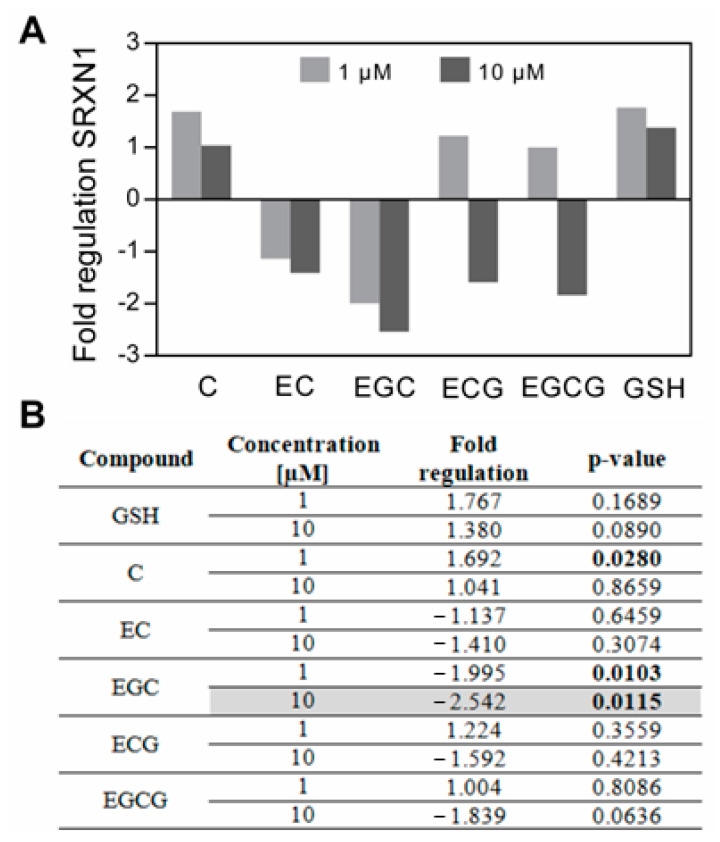
Catechins affect *SRXN1* transcription. (**A**) Changes in the expression of the SRXN1 gene induced by 24 h treatment of the HT29 cell line with catechins or glutathione seemed to be structure dependent. (**B**) Statistical significance of the data presented in Figure 1A. Statistically significant and biologically relevant changes in *SRXN1* expression have been bolded and highlighted, respectively. The results are means of three biological replicates. Abbreviations: C, (+)-catechin; EC, (−)-epicatechin; EGC, (−)-epigallocatechin; ECG, (−)-epicatechin gallate; EGCG, (−)-epigallocatechin gallate; GSH, glutathione.

Catechins are known to affect the expression of genes implicated in the maintenance of cellular redox homeostasis, such as these encoding phase II enzymes [28]. However, relatively less information is on *SRXN1*. Our former microarray analysis of the expression of redox-related genes showed that EGC at a relatively high concentration (10 µM) caused a statistically significant (*p* < 0.05, fold change > 2) drop in SRXN1 gene transcription in HT29 cells [9]. The trends in the modulation of SRXN1 gene transcription observed for treatment with other catechins seemed to be influenced by their chemical structures. These structure–activity relationships are illustrated in Figure 1A. The parent form, i.e., (+)-catechin (C), was able to increase *SRXN1* expression. Epimers of C, (−)-epicatechin (EC) and (−)-epigallocatechin (EGC), caused a decrease in *SRXN1* transcription, whereas esters of catechin and gallic acid, (−)-epicatechin gallate (ECG) and (−)-epigallocatechin gallate (EGCG), increased *SRXN1* expression at lower concentration (1 µM), but decreased it when applied at higher concentration (10 µM). In contrast, GSH, a major intracellular antioxidant, exhibited no significant stimulatory impact on *SRXN1* transcriptional activity when applied exogenously to HT29 cells (Figure 1B). Here, we examined if DNA methylation could be one of the potential mechanisms responsible for the downregulation of *SRXN1* expression and how this phenomenon relates to the physicochemical properties of investigated compounds.

### 3.2. Methylation of SRXN1 Promoter

**Figure 2:**
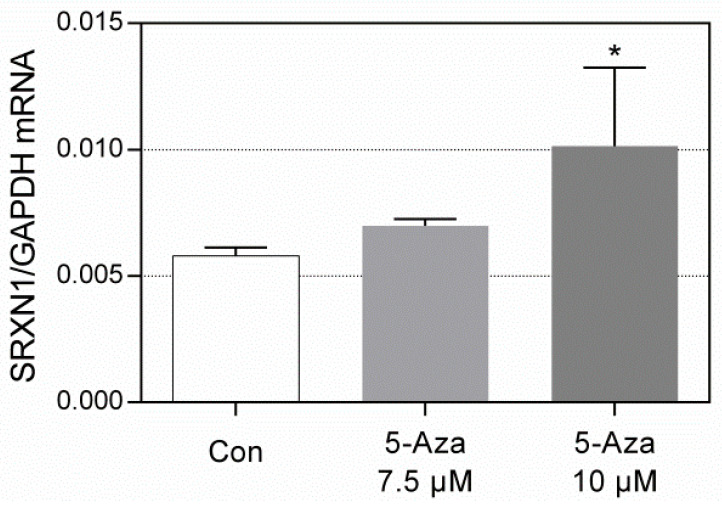
*SRXN1* transcription can be modulated by DNA methylation as illustrated by the changes in the mRNA expression level of the SRNX1 gene estimated by RT-qPCR after 72 h treatment of HT29 cells with 7.5 or 10 µM 5-azacitidine (5-Aza). The results are means ± SD of three biological replicates. Statistical analysis was carried out with one-way ANOVA with Dunnett’s test; (*)—*p* ≤ 0.05. Abbreviations: C, (+)-catechin; Con, control; EC, (−)-epicatechin; EGC, (−)-epigallocatechin; ECG, (−)-epicatechin gallate; EGCG, (−)-epigallocatechin gallate; GSH, glutathione.

To examine whether mRNA expression of *SRXN1* could be regulated by DNA methylation, HT29 cells were treated for 72 h with DNA demethylating agent 5-azacytidine (5-Aza) in concentrations of 7.5 µM and 10 µM, which for the treatment of HT29 cells with this compound corresponded to EC_30_ and EC_45_, respectively (Supplementary Materials Figure S1). After treatment with a lower 5-Aza concentration, RT-qPCR revealed the same level of SRXN1 mRNA as in control cells. In contrast, a statistically significant, almost 2-fold increase in SRXN1 mRNA level was determined after treatment with 10 µM 5-Aza (Figure 2). These results confirmed that, indeed, DNA methylation may be an important regulator of SRXN1 gene transcription.

**Figure 3:**
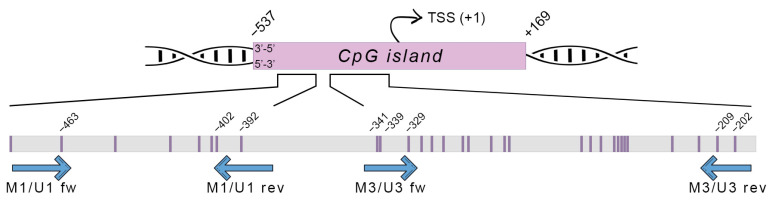
Scheme of the investigated CpG island encompassing the promoter region of the SRXN1 gene. Primer binding sites are shown in the inset with CpG dinucleotides marked as vertical lines. The position of the transcription starting site (TSS) is marked as “+1”; M1/U1 fw, M3/U3 fw: methylated and unmethylated forward primers, respectively; M1/U1 rev, M3/U3 rev: methylated and unmethylated reverse primers, respectively.

The gene for SRXN1 is located on the minus strand of chromosome 20, extending from position 646,615 to 653,200 (NCBI RefSeq NC_000020.11). According to the EMBOSS CpGplot, the human *SRXN1* contains a 706 bp long CpG island that covers the region from 652,858 to 653,032, i.e., −537 to +169, with regard to the position of the transcription start site (TSS) marked as +1 (NCBI RefSeq NM_080725.3) (Figure 3). In order to evaluate the effect of catechins on the DNA methylation profile of CpG island within the promoter of the SRXN1 gene, MSP analysis of one (M1/U1) and HRM analysis of two selected regions (M1/U1 and M3/U3) were performed. Positions of the primers used for DNA methylation analysis are shown in Figure 3.

#### 3.2.1. DNA Methylation within M1/U1 Region of *SRXN1* Promoter Analyzed by MSP

**Figure 4:**
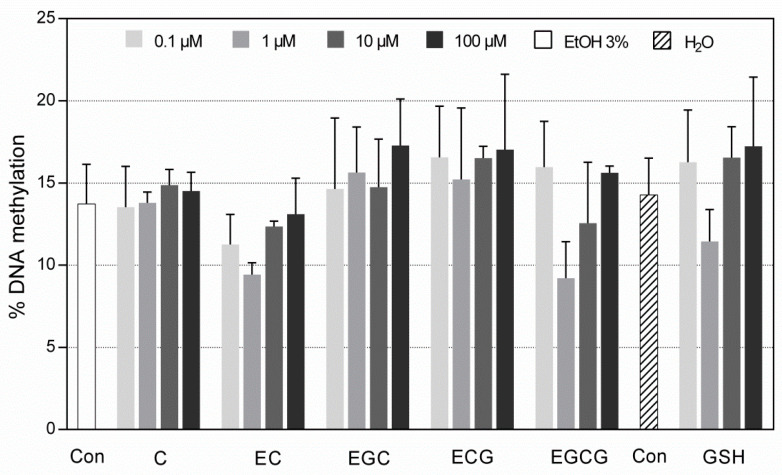
Changes in DNA methylation of specific CpGs induced in HT29 cells by catechins at 0.1–100 µM concentrations. Relative methylation level of the primer-binding sequence in the *SRXN1* promoter region was investigated by methylation-specific PCR (MSP) with M1/U1 primer sets (M1/U1, methylated and unmethylated primer, respectively). The results are means ± SD of three biological replicates. No statistically significant changes were detected by one-way ANOVA with Dunnett’s test. Abbreviations: C, (+)-catechin; Con, control; EC, (−)-epicatechin; EGC, (−)-epigallocatechin; ECG, (−)-epicatechin gallate; EGCG, (−)-epigallocatechin gallate; GSH, glutathione.

MSP analysis with M1/U1 primer pairs showed some trends but no statistically significant differences in DNA methylation level between any of the treatments compared to the control (Figure 4). In the case of HT29 cells treated with C, DNA methylation remained at the same level as in control cells, while treatment with EC tended to decrease DNA methylation level, especially at the concentration of 1 µM, where a decline of 31.3% was observed. In other groups of treated cells, a slight increase in DNA methylation was detected with all applied concentrations, except in the cells treated with 1 µM and 10 µM EGCG. The highest increase was observed in cells treated with the highest concentrations of ECG (up to 24.1%) and EGC (up to 25.9%), and indeed, the latter compound downregulated *SRXN1* significantly (Figure 1. GSH at concentrations ranging from 0.1 µM to 100 µM tended to increase DNA methylation level from 14% to 20.7%, respectively, compared to control, with the exception of 1 µM GSH-treated cells, where 19.8% decrease in DNA methylation was observed (Figure 4). The percentage of DNA methylation, fold-changes, and *p*-values obtained by MSP analysis are presented in Supplementary Materials (Supplementary Materials Table S3). The obtained MSP results suggest that CpGs at positions 463, −402, and −392 (present in the DNA sequence complementary to the primer sequences) were not differentially methylated in cells treated with different catechins compared to the control.

#### 3.2.2. DNA Methylation in Both M1/U1 and M3/U3 Regions of *SRXN1* Promoter Analyzed by MS-HRM

**Figure 5:**
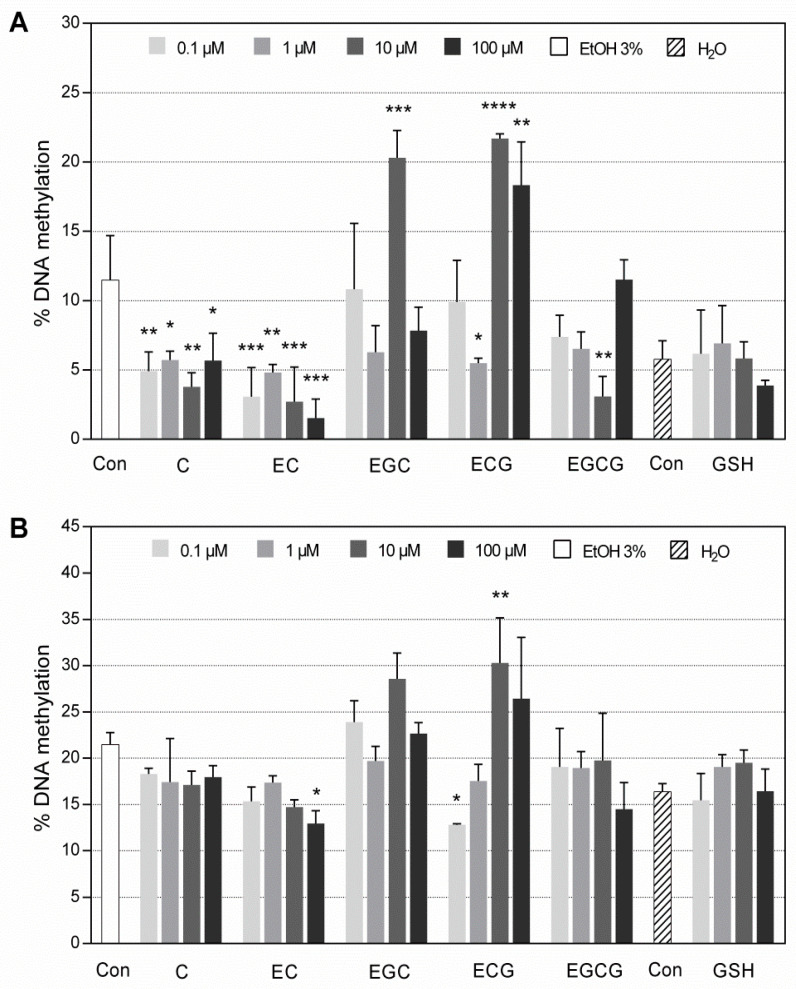
Changes in the methylation levels of the selected *SRXN1* promoter region induced in HT29 cells by 24 h treatment with catechins or glutathione at 0.1–100 µM concentrations. The selected area of the CpG island of the SRXN1 gene promoter was investigated by methylation-sensitive high-resolution melting (MS-HRM) with the use of either M1/U1—(**A**) or M3/U3—(**B**) primers, where M1/U1 and M3/U3 refer to methylated and unmethylated primers, respectively. The results are means ± SD of three biological replicates. Statistical analysis was performed using one-way ANOVA with Dunnett’s test. The asterisks mark *p*-values as follows: (*)—≤ 0.05; (**)—≤ 0.01, (***)—≤ 0.001, and (****)—≤ 0.0001. Abbreviations: C, (+)-catechin; Con, control; EC, (−)-epicatechin; EGC, (−)-epigallocatechin; ECG, (−)-epicatechin gallate; EGCG, (−)-epigallocatechin gallate; GSH, glutathione.

To include an additional four CpGs in DNA methylation analysis, positioned in the region between forward and reverse primer pairs, HRM analysis of the same region as for MSP was conducted with a mix of four primers (M1_fw_/M1_rev_ and U1_fw_/U1_rev_). This region covered the sequence from −482 to −378 with regard to the position of TSS. In that way, detailed information regarding the DNA methylation status of the targeted *SRXN1* region (7 CpGs in total) was obtained. Analysis showed a statistically significant decrease in DNA methylation level in C- and EC-treated cells compared to control cells at all applied concentrations (decrease up to 86.7%, Figure 5A). Additionally, the diminishment of DNA methylation level was statistically significant also for HT29 cells treated with 1 µM ECG (52.2%) and 10 µM EGCG (73.1%). The increase of DNA methylation level was observed only in cells treated with 10 µM EGC as well as 10 µM and 100 µM ECG. DNA methylation levels were 76.8%, 88.9%, and 59.6% higher than under control conditions, respectively, and the differences were statistically significant. Other applied concentrations of catechins and treatments with GSH did not influence the *SRXN1* DNA methylation status compared to adequate control (Figure 5A). The percentage of DNA methylation, fold-changes, and *p*-values obtained as a result of HRM analysis are presented in Supplementary Materials (Table S4). For treatments that caused a statistically significant increase in DNA methylation, representative aligned melt curves, difference plots showing positions of control and catechin curves with respect to 0%, 50%, and 100% methylated standards, standard curves, and bar graphs representing DNA methylation levels obtained from standard curves are separately presented in Supplementary Materials Figure S2.

Another region of *SRXN1* promoter was analyzed by MS-HRM with a mix of four primers (M3_fw_/M3_rev_ and U3_fw_/U3_rev_) to estimate changes of DNA methylation of 23 CpGs located within the sequence from −345 to −190 directly upstream of the TSS. The changes in the methylation levels are shown in Figure 5B. As in the first analyzed region, there was a decline in DNA methylation level in HT29 cells treated with all concentrations of C and EC, compared to control, still with statistical significance achieved only in the case of cells treated with 100 µM EC (39.8%). Additionally, a significant decrease in DNA methylation was also observed in 0.1 µM ECG-treated cells (40.4%). HRM analysis showed more than a 20% increase in DNA methylation level in cells treated with 10 µM EGC and 10 µM and 100 µM ECG among which the treatment with 10 µM ECG showed a statistically significant increase (41%) compared to the control. In other applied concentrations and treatments, including GSH, DNA methylation levels were comparable to adequate controls (Figure 5B). The percentage of DNA methylation, fold-changes, and *p*-values obtained as a result of HRM analysis are presented in Supplementary Materials (Table S4). Representative aligned melt curves, difference plots, standard curves, and bar graphs are separately presented for treatments that caused a statistically significant increase in DNA methylation in Supplementary Materials Figure S3.

### 3.3. RT-qPCR Analysis of SRXN1 Expression

**Figure 6:**
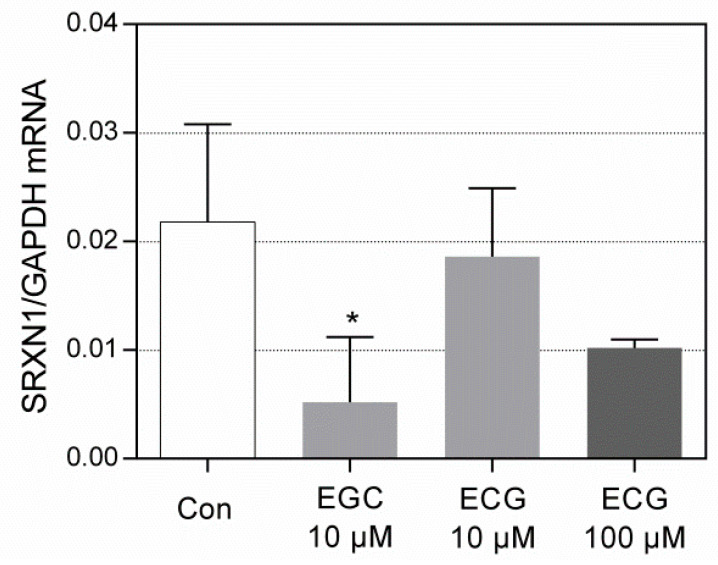
Downregulation of *SRXN1* following increased DNA methylation is induced only by (−)-epigallocatechin (EGC). Changes in mRNA expression level estimated by RT-qPCR for SRXN1 gene after 24 h treatment of HT29 cells with 10 µM EGC, 10 µM, and 100 µM (−)-epicatechin gallate (ECG). The results are means ± SD of three biological replicates. Statistical analysis was carried out by one-way ANOVA with Dunnett’s test; (*)—*p*-value ≤ 0.05. Con refers to control treatment.

To confirm that the observed changes in DNA methylation levels are associated with altered levels of SRXN1 mRNA expression, RT-qPCR was performed for the treatments that showed a statistically significant increase in DNA methylation level in the case of HRM analysis (Figure 5). In HT29 cells treated with 10 µM ECG, SRXN1 mRNA expression was 14.5% lower than in control, while in cells treated with 100 µM ECG, this decline reached 53.3% compared to control. The lowest and statistically significant decrease in SRXN1 mRNA expression was observed in HT29 cells treated with 10 µM EGC (Figure 6). Downregulation of 76.2% compared to the control SRXN1 mRNA level was in accordance with the results obtained from profiler analysis (Supplementary Materials Table S5).

### 3.4. The Search for Correlations between Investigated Parameters

The correlation between levels of DNA methylation and available parameters defining redox properties for both catechins and GSH, as well as their bioactivity (gathered in Table 1), was investigated by linear methods: *t*-test, Welch’s test, and Cochran test. In this case, we compared the results of DNA methylation levels performed by the MS-HRM method for the M1/U1 pair of primers and each individual electrochemical parameter, as presented in Table 1. No significant correlation was found for any of these parameters. However, all tests applied suggested some hypothetical influence of *Q_ox_*, *AOE*, and *t* because, in these cases, *p*-values in the *t*-test were *p* ≤ 0.15. In other cases, the *t*-test showed *p* > 0.18 or much higher. Therefore, these three electrochemical parameters were analyzed for possible strong interactions. For this purpose, again, the three abovementioned statistical tests were applied to investigate nonlinear effects. This approach revealed the impact on DNA methylation of strong interactions between *Q_ox_*_._ and *t* (*t*-test *p* < 0.0380, Welch’s test *p* < 0.0271, Cochrane test *p* < 0.0999). The interactions between *AOE* and *t* did not reach statistical significance (*t*-test *p* < 0.0831, Welch’s test *p* < 0.1009, Cochrane test *p* < 0.1969) and thus seemed to have a smaller impact.

## 4. Discussion

Epigenetic mechanisms affect various biological processes involved in the preservation of health as well as implicated in the initiation and progression of diseases. Epigenetic modifications, e.g., DNA methylation, are known to be reversible in response to various environmental factors, including diet. Therefore, it is not surprising that the investigations on epigenetic factors behind the beneficial activities of dietary compounds have gained much attention. The impact of food components on DNA methylation can occur at four levels: (i) availability of methyl donors, (ii) modulation of DNMTs activity, (iii) impact on the activity of enzymes involved in one-carbon metabolism, and (iv) involvement in mechanisms related to active DNA demethylation [31]. Methylation of CpGs in DNA is maintained by DNMT-dependent transfer of methyl groups from SAM, a methyl donor generated in the methionine cycle [32]. The methionine cycle is closely related to the folate cycle, and together, these pathways shape the “one-carbon metabolism”. Numerous nutrients and some non-nutritive compounds (e.g., catechins) have been reported to affect one-carbon metabolism and subsequent SAM generation, which further leads to the modulation of histone and DNA methylation levels [33]. To date, catechins have been demonstrated to affect DNA methylation by interfering with folic acid metabolism as well as by inhibiting of DNMTs activity and expression [10,34]. Even though it is generally agreed that catechins mainly act as direct DNMT1 inhibitors, in mice whose diet was supplemented with EGCG, the decrease in this enzyme activity was accompanied by the lowered expression of the DNMT1 gene caused by the increased level of DNA methylation in its promoter [35]. These latter results suggest that catechins may exhibit the ability to influence DNA methylation also by mechanisms other than inhibition of DNMT1 enzymatic activity.

Previously, we had reported a rather surprising observation that one catechin, namely EGC, downregulates the expression of the SRXN1 gene in human colon HT29 cells [9]. Therefore, in the current study, we investigated using the same cell line whether the observed EGC-induced decrease in *SRXN1* transcription is associated with changes in its promoter methylation pattern, similarly as it was shown for EGCG and *DNMT1* expression [35]. The initial assumption implied that the changes in expression of the SRXN1 gene might be a consequence of epigenetic modulation triggered by an altered redox environment. This assumption stemmed from former results suggesting that in HT29 cells, catechins at 10 µM concentration brought the cellular redox status to the borderline of maintained homeostasis [9]. Recently it has been shown that a sustained overexpression of nuclear factor, erythroid 2-like 2 (Nrf2)-driven antioxidant transcriptome (involving *SRXN1*) leads to reductive stress in cardiac tissue *in vivo* [36], whereas catechins are known modulators of Nrf2 expression [37]. We hypothesized that in cells exposed to strong antioxidants, the production of antioxidant enzymes is not needed, and as a result, the silencing of expression of some genes, e.g., of *SRXN1*, via DNA methylation takes place to maintain proper redox homeostasis, in particular, to prevent pushing cells into reductive stress. To verify this hypothesis, the impact of catechins on the DNA methylation profile of the SRXN1 gene in HT29 cells was investigated in a wide concentration range, from 0.1 µM physiologically to 100 µM intestinally achievable, to pinpoint the possible dose–response relationships for a series of catechin derivatives differing in chemical structures and electrochemical properties.

The results of the MS-HRM study confirmed our hypothesis that the observed EGC-induced downregulation of *SRXN1* expression (Figure 1 and Figure 6) may be related to changes in the DNA methylation of this gene promoter. However, the clear cause–effect relationship for antioxidants studied turned out to be difficult to pinpoint at first sight, which is not surprising, taking into account the variety of mechanisms influencing DNA methylation triggered by these polyphenols. The level of DNA methylation within the first analyzed region of the *SRXN1* promoter significantly increased in the case of treatment of HT29 cells with high concentrations of EGC and ECG. As mentioned before, high concentrations of catechins are relevant only to colonic cells, which are in direct contact with ingested food [38]. The ileal fluid reaching the colon may contain up to 70% of ingested catechins [39]; thus, their effective concentrations exceed those found in human plasma that reache around 1 µM if these polyphenols are consumed in typical amounts [40]. In our hands, EGC and ECG applied to HT29 cells at physiological concentrations either did not affect or slightly decreased DNA methylation in the *SRXN1* promoter. For C and EC, a decrease in DNA methylation level was observed, regardless of the applied concentration (Figure 5). Although GSH was reported to be involved in the regulation of several epigenetic mechanisms, it hardly influenced the DNA methylation level of any of the analyzed regions, which is in line with earlier studies reviewed by García-Giménez et al. [41]. Moreover, recently endogenous reducing thiols, including GSH, have even been shown to support SRXN1-driven reactivation of PRDXs [42], which may explain why this thiol antioxidant should not be expected to downregulate *SRXN1* expression.

The chemical structures of EGC and ECG that increased DNA methylation of SRXN1 gene promoter do not possess any specific structural features that are not seen in other catechin derivatives. The *cis* configuration of substituents on carbons C2 and C3, which shapes the 3-dimensional structure of the whole molecule, and hence could impact interaction with target proteins, is also present in other derivatives (except for C being the *trans* epimer). The hydrophobicity of ECG is higher than that of EC but lower than that of EGCG [43]. Both latter compounds, in contrast to ECG, decrease rather than increase DNA methylation. The presence of pyrogallol moiety also does not seem to have a decisive role, as EGCG, which did not increase DNA methylation level, contains two such substituents. Moreover, all studied pyrogallol derivatives display similar chelating properties [30], i.e., the feature that could alter their availability for interactions. In addition, the ability to form internal hydrogen bonds is similar in the case of both ECG and EGCG [30]. All mentioned facts make it impossible to propose any straightforward structure–activity relationship and to indicate any sole physicochemical property of catechins as that influencing cellular DNA methylation machinery.

Moreover, the treatment of HT29 cells with different concentrations of catechins did not reveal any clearcut concentration dependence for SRXN1 gene promoter DNA methylation. However, a similar lack of concentration dependence was observed for the same set of catechins and their ability to influence the expression of redox-related genes in HT29 cells in our earlier nutrigenomic experiments [9]. Such observations may suggest that there are additional intracellular factors/mechanisms that somehow counterbalance the biological actions of catechins. One such mechanism may be related to their metabolism, in particular, the pathway of biotransformation of catechins upon which these compounds become methylated [44]. As already mentioned, catechins are methylated by COMT, which competes with DNMTs for methyl groups provided by SAM [16]. Thus, COMT-catalyzed reactions decrease the pool of available SAM and increase its demethylated form, SAH, which may further inhibit DNMT1 [17]. Such a mechanism could explain the decrease in DNA methylation observed in the case of treatment of HT29 cells with catechol moiety containing C and EC, but not the impact of pyrogallol derivatives of epicatechin on methylation of *SRXN1* promoter (Figure 5A).

**Figure 7:**
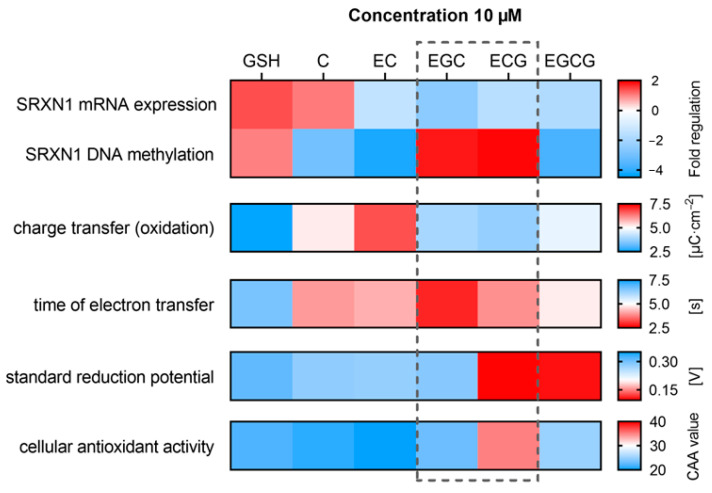
Heatmap representing an overview of chemical parameters of studied catechins and their effects on cellular antioxidant activity, SRXN1 mRNA expression level, and DNA methylation of *SRXN1* promoter region. Abbreviations: C, (+)-catechin; CAA, cellular antioxidant activity; EC, (−)-epicatechin; EGC, (−)-epigallocatechin; ECG, (−)-epicatechin gallate; EGCG, (−)-epigallocatechin gallate; GSH, glutathione.

Other analyzed features of compounds studied, which via influencing cellular redox status, could affect DNA methylation level, included their redox properties. The way of reasoning here was the following: (i) active demethylation occurs as a result of oxidation of methyl group in 5-methylcytosine [45], (ii) strong antioxidants scavenge ROS creating a more reductive cellular environment, (iii) under such conditions, active DNA demethylation becomes less probable, (iv) as a result DNA methylation levels may increase. Reduction potentials represent the electrochemical property that may thus determine the impact of antioxidants on cellular redox homeostasis. Initially, we assumed that this property of catechins was behind the increased DNA methylation and, therefore, lower expression of the SRXN1 gene incurred by some catechins [9]. We speculated that the ease of donating electrons by these antioxidant compounds drives the cellular environment toward a more reductive state, thereby preventing active DNA demethylation. In general, thermodynamically, the lower the standard reduction potential *E^0^*, the higher the antioxidant activity, which in the case of the studied redox-active compounds increases at 37 °C in the following order: GSH < EGC < C < EC < EGCG < ECG [9]. The presented results seemed to clearly disprove this initial working hypothesis. The increase in DNA methylation was induced by EGC and ECG, which, among other catechins, displayed the lowest and the highest standard reduction potentials, respectively. GSH, used as a reference antioxidant, did not affect DNA methylation within the examined promoter area of *SRXN1* regardless of its concentration, even though its standard reduction potential is similar to that of EGC. Thus, the observed changes in the DNA methylation level of this gene did not seem to depend on the reduction potentials of investigated compounds. However, the total antioxidant activity (TAA) of a compound is determined not only by thermodynamics, reflected by a standard reduction potential. In addition, the kinetics of oxidation of an antioxidant, expressed as the charge transferred in the oxidation reaction within a time unit, influences the TAA. The implementation of a kinetic factor in antioxidant activity determination, which previously was reported for catechins as the stoichiometry value n_10_, showed that the total reducing capacity of EGC was stronger than it appeared based solely on *E^0^* (Figure 7) [9]. Indeed, our preliminary results of voltammetric measurements performed for catechins revealed that during the oxidation process, EGC transferred about twice the amount of charge compared to that transferred by ECG within a similar time period (Table 1). Due to the lack of complete electrochemical characterization of catechins, this aspect simply could not be satisfactorily clarified. Nonetheless, the importance of kinetic aspects was confirmed by the applied statistical tests investigating nonlinear effects, which revealed the impact of strong interactions between *Q_ox_*_._ and *t* on the DNA methylation of *SRXN1* promoter (Section 3.4), and maybe also other redox-related albeit not yet identified genes, in cells exposed to strong antioxidants.

Altogether, our results that compare redox properties of studied catechins with their impact on DNA methylation summarized graphically in the heatmap included in Figure 7, point to the fact that the effectiveness of reducing (exogenous) compounds in shaping cellular redox status may depend on both thermodynamics and kinetics of redox processes, in which they are involved. It can be noticed that both EGC and ECG differ from other catechins in two features: (i) medium ability to transfer charge, but (ii) in the shortest times. This makes them very efficient antioxidants. To maintain proper homeostasis, cells must neutralize oxidants by mobilization of an antioxidant defense system but also must respond to particularly effective reducing agents that could block cellular ROS-dependent processes [22]. In the latter case, lowering expression of *SRXN1* by DNA methylation of its promoter seems a particularly powerful mechanism as this enzyme is necessary for the restoration of activity of one of the most important classes of antioxidant enzymes—peroxiredoxins as well as the reduction of other numerous S-sulfinylated proteins [46]. Our study provides the unique proof-of-concept that changes in the DNA methylation profile of a redox-related gene promoter, thus biological effects, may be affected by the electrochemical properties of antioxidants shaping cellular redox homeostasis. In view of the fact that our reasoning focused mainly on the chemical explanation of the observed biological effects and concentrated on one specific group of polyphenols, more research is needed to confirm that the proposed mechanism of modulation of redox homeostasis can also be observed in the case of other reducing agents or antioxidant-related genes or other cellular models.

## 5. Conclusions

In conclusion, our study demonstrated that the downregulation of SRXN1 gene transcription by some catechins might be a result of increased DNA methylation level within its promoter. This effect seems to depend neither on the standard reduction potentials of these antioxidants nor on their chemical structures as decisive features but rather on the kinetics of redox reaction. A comprehensive electrochemical characterization of catechins and more advanced dedicated experiments are needed to fully understand whether the changes in DNA methylation level in the *SRXN1* promoter might have been driven by the postulated redox-sensitive cellular response. The demonstrated increased DNA methylation of *SRXN1* promoter region by EGC and ECG sheds new light on the epigenetic potential of catechins, which so far has been mainly associated with the impaired maintenance of global DNA methylation patterns as a result of DNMT1 activity inhibition. Our results revealed unexpected interrelations between otherwise seemingly independent phenomena (electrochemistry and epigenetics), indicating that novel, interdisciplinary research approaches are needed to define the cause-and-effect relationship for bioactive food components before their usefulness in epigenetic-based cancer chemoprevention becomes predictable.

## Figures and Tables

**Table 1:** Summary of data concerning chemical and electrochemical parameters as well as biological effects of catechins.

Compound	GSH	C	EC	EGC	ECG	EGCG	Ref.
Structure	Tripeptide	Parent compound	Epimer of C	Epimer of GC	Ester of EC with gallate	Ester of EGC with gallate	[9]
Catechol moiety	0	1	1	0	1	0	
Pyrogallol moiety	0	0	0	1	0	1	
Galloyl moiety	0	0	0	0	1	1	
**Cell-free systems**							
**Electrochemical parameters**							
*E^0^* at 37 °C (V)	0.310 ± 0.003	0.281 ± 0.008	0.277 ± 0.005	0.287 ± 0.003	0.098 ± 0.002	0.104 ± 0.002	[9]
*E_p,a_ + ɛ* at 25 °C (V)	0.984 ± 0.000	0.273 ± 0.000	0.260 ± 0.007	0.140 ± 0.007	0.230 ± 0.007	0.120 ± 0.009	[29]
*I_p,a_* at 25 °C (µA)	2.60 ± 0.17	5.40 ± 0.38	8.10 ± 0.28	12.60 ± 0.26	5.70 ± 1.06	6.30 ± 0.63	
*Q_ox._* at 25 °C (µC·cm^−2^)	2.6 ± 0.08	5.20 ± 0.05	6.70 ± 0.10	3.90 ± 0.04	3.70 ± 0.65	4.70 ± 0.35	
*AOE* (µJ·cm^−2^)	233.3 ± 3.50	184.40 ± 4.42	227.00 ± 1.40	65.50 ± 0.32	113.80 ± 13.83	119.30 ± 7.86	
*t* (s)	6.6 ± 0.0	4.0 ± 0.0	4.2 ± 0.0	2.9 ± 0.0	3.9 ± 0.0	4.8 ± 0.0	
*AOP* (µW·cm^−2^)	35.4 ± 0.53	46.10 ± 1.10	54.10 ± 0.34	22.60 ± 0.11	29.20 ± 3.55	24.90 ± 1.64	
**Antioxidant activity by spectrophotometric tests**							
Fe(III) chelation, pFe pH 7.0	-	17.6 *	-	23.1	23.2	23.4	[30]
FRAP (mol TE/mol), pH 3.6	0.01 ± 0.00	0.79 ± 0.00	0.92 ± 0.00	1.03 ± 0.01	2.34 ± 0.01	2.21 ± 0.01	[2]
**Spectro. test**	**Antioxidant activity, n_10_ value at 37 °C**						
ABTS	1.52 ± 0.02	5.44 ± 0.07	5.44 ± 0.11	5.98 ± 0.10	7.76 ± 0.11	8.33 ± 0.09	[9]
DPPH	0.01	2.15 ± 0.03	2.20 ± 0.03	3.02 ± 0.03	5.48 ± 0.07	6.16 ± 0.08	
FC	0.39	1.96 ± 0.02	1.83 ± 0.03	1.50 ± 0.02	2.66 ± 0.02	2.31 ± 0.03	
**Protection against oxidation ^a^/fluorescein bleaching ^b^ expressed as IC_50_ (µM)**							
DHR123 ^a^	14.07 ± 0.36	0.81 ± 0.07	1.36 ± 0.04	1.21 ± 0.05	1.08 ± 0.04	1.15 ± 0.00	[2]
NaOCl ^b^	3.52 ± 0.05	0.34 ± 0.00	0.51 ± 0.03	0.25 ± 0.03	0.31 ± 0.01	0.36 ± 0.01	
AAPH ^b^	15.44 ± 0.01	0.67 ± 0.04	1.08 ± 0.06	0.66 ± 0.03	1.87 ± 0.07	2.16 ± 0.17	
**Cell culture**							
**Conc.**	**Cell growth (% of control) at 37 °C, 24 h**						
1 µM	94.3 ± 5.8	109.7 ± 10.8	127.8 ± 10.2	97.7 ± 9.3	110.5 ± 11.3	94.3 ± 5.8	[9]
10 µM	90.8 ± 4.3	96.1 ± 9.9	106.5 ± 7.8	92.9 ± 10.4	101.2 ± 10.6	90.8 ± 4.3	
**Conc.**	**Cellular antioxidant activity, CAA value at 37 °C, 1 h**						
1 µM	7 ± 7	11 ± 8	23 ± 3	17 ± 5	35 ± 9	22 ± 10	[9]
10 µM	22 ± 6	21 ± 10	20 ± 7	23 ± 9	35 ± 9	25 ± 10	
**Conc.**	**Genotoxicity % at 37 °C, 24 h**						
1 µM	6.75 ± 0.88	3.58 ± 0.29	3.07 ± 0.23	3.54 ± 0.05	2.92 ± 0.69	2.59 ± 0.81	[9]
10 µM	6.35 ± 2.01	3.77 ± 0.62	3.04 ± 1.00	3.02 ± 0.37	2.55 ± 0.35	2.66 ± 0.63	
**Conc.**	**Fold regulation of gene expression at 37 °C, 24 h**						
1 µM	1.8	1.7	−1.1	−2.0	1.2	1.0	[9]
10 µM	1.4	1.0	−1.4	−2.5	−1.6	−1.8	
**Conc.**	**Fold change of % DNA methylation at 37 °C, 24 h**						
	**MSP M1/U1**						Current study
1 µM	0.802 ± 0.136	1.005 ± 0.047	0.687 ± 0.053	1.139 ± 0.202	1.109 ± 0.317	0.671 ± 0.162	
10 µM	1.159 ± 0.131	1.084 ± 0.069	0.900 ± 0.024	1.074 ± 0.213	1.204 ± 0.052	0.915 ± 0.269	
**Conc.**	**MS-HRM M1/U1**						
1 µM	1.199 ± 0.471	0.498 ± 0.054	0.419 ± 0.051	0.547 ± 0.167	0.478 ± 0.032	0.569 ± 0.106	
10 µM	1.009 ± 0.208	0.330 ± 0.088	0.236 ± 0.217	1.768 ± 0.171	1.889 ± 0.029	0.269 ± 0.126	
**Conc.**	**MS-HRM M3/U3**						
1 µM	1.163 ± 0.080	0.811 ± 0.219	0.809 ± 0.034	0.918 ± 0.073	0.816 ± 0.084	0.882 ± 0.082	
10 µM	1.191 ± 0.084	0.797 ± 0.070	0.685 ± 0.037	1.331 ± 0.130	1.410 ± 0.227	0.920 ± 0.236	

* Water, exact pH not specified. Abbreviations: AAPH, 2,2′-azobis(2-amidinopropane) dihydrochloride; ABTS, test employing 2,2′-azino-bis(3-ethylbenzothiazoline-6-sulfonic acid) radical cation; *AOE*, specific antioxidant energy; *AOP*, antioxidant power; C, (+)-catechin; DHR123, dihydrorhodamine 123; DPPH, test employing 1-diphenyl-2-picrylhydrazyl radical; EC, (−)-epicatechin; EGC, (−)-epigallocatechin; ECG, (−)-epicatechin gallate; EGCG, (−)-epigallocatechin gallate; FC, test employing Folin–Ciocalteu reagent; FRAP, ferric reducing antioxidant power assay; GC, (−)-gallocatechin; GSH, glutathione; *E^0^*, standard reduction potential; *E_p,a_ + ɛ*, peak potential versus standard hydrogen electrode; *I_p,a_*, anodic current; NaOCl, sodium hypochlorite; *Q_ox_*, charge transfer in oxidation process; *t*, time of electron transfer.

## Data Availability

The data supporting reported results can be found in Supplementary Materials.

## Supplementary Materials

The following supporting information can be downloaded at: https://www.mdpi.com/article/10.3390/antiox12030754/s1, Figure S1: Inhibition of growth of HT29 cells determined by MTT assay after 72 h treatment with 5-Aza; Figure S2: The increase in DNA methylation level of SRXN1 gene determined by MS-HRM using M1/U1 primer sets following 24 h treatment of HT29 cells with catechins that increase methylation profile of SRXN1 promoter; Figure S3: The increase in DNA methylation level of SRXN1 gene determined by MS-HRM using M3/U3 primer sets following 24 h treatment of HT29 cells with catechins that increase methylation profile of SRXN1 promoter; Table S1: Primer sequences for SRXN1 gene used in MSP and MS-HRM; Table S2: Primer sequences for SRXN1 and GAPDH genes used in RT-qPCR; Table S3: Methylation changes in CpG island within promoter region of SRXN1 in HT29 cell line investigated with MSP; Table S4: Methylation changes in CpG island within promoter region of SRXN1 in HT29 cell line investigated with MS-HRM; Table S5: Changes in SRXN1 gene expression analysed with RT-qPCR method.
