# Supplementary material for: Beyond Antioxidant Activity: Redox Properties of Catechins May Affect Changes in the DNA Methylation Profile—The Example of SRXN1 Gene
Source: Antioxidants (Basel). 2023 Mar 20;12(3):754. doi: 10.3390/antiox12030754 (PMC10045509; doi:10.3390/antiox12030754)
Supplement: Supplementary file 1 [file antioxidants-12-00754-s001.zip › antioxidants-2257440-supplementary.pdf]

SUPPLEMENTARY MATERIALS

Table S1. Primer sequences for SRXN1 gene used in MSP and MS-HRM.

**Primers**	**Product size**	**Forward 5'→3'**	**Reverse 5'→3'**
M1	103 bp	GTTAGATTGGAAGTGGAATCGTT	CCAAAATAAATCGACAAAACCC
U1	105 bp	GTTAGATTGGAAGTGGAATTGT	AACCAAAATAAATCAACAAAACCC
M3	156 bp	TTTTCGCGGTTTAAGTCGGT	AAACTCTCCACCGAAAAACG
U3	151 bp	GTTTTTGTGGTTTAAGTTGGTT	TCCACCAAAAAACACCTCTC

Table S2. Primer sequences for SRXN1 and GAPDH genes used in RT-qPCR.

**Gene**	**Product size**	**Forward 5'→3'**	**Reverse 5'→3'**
SRXN1	104 bp	AACTAGCTGGACCCGTCACC	TCGGGCCAAGGGCATCTAAG
GAPDH	131 bp	GTCTCCTCTGACTTCAACAGCG	ACCACCCTGTTGCTGTAGCCAA

Table S3. Methylation changes in CpG island within promoter region of *SRXN1* in HT29 cell line investigated with MSP. Cells were treated with catechins and glutathione at concentrations of 0.1, 1, 10 and 100 µM for 24 h at 37°C. The results are means ± SD of three biological replicates.

**Compound**	**Concentration [µM]**	**MSP M1/U1**		
		% DNA methylation	Fold change	*p*-value*
C	0.1	13.53	0.986	0.9999
	1	13.80	1.005	>0.9999
	10	14.87	1.084	0.9993
	100	14.51	1.057	0.9995
EC	0.1	11.25	0.820	0.9577
	1	9.43[a]	0.687	0.5784
	10	12.35	0.900	0.9991
	100	13.11	0.955	0.9996
EGC	0.1	14.63	1.066	0.9994
	1	15.64	1.139	0.9909
	10	14.74	1.074	0.9994
	100	17.29	1.259	0.6757
ECG	0.1	16.56	1.207	0.8919
	1	15.22	1.109	0.9990
	10	16.52	1.204	0.9018
	100	17.03	1.241	0.7617
EGCG	0.1	15.97	1.163	0.9818
	1	9.21	0.671	0.3687
	10	12.56	0.915	0.9993
	100	15.62	1.138	0.9912
GSH	0.1	16.26	1.140	0.8034
	1	11.44	0.802	0.5787
	10	16.54	1.159	0.7297
	100	17.23	1.207	0.5431

* statistical analysis was performed using one-way ANOVA with Dunnett's test with a cut at $p < 0.05$

[a] the result is a mean ± SD of two biological replicates

Table S4. Methylation changes in CpG island within promoter region of *SRXN1* in HT29 cell line investigated with MS-HRM. Cells were treated with catechins and glutathione at concentrations of 0.1, 1, 10 and 100 µM for 24 h at 37°C. The results are means ± SD of three biological replicates.

Compound	Concentration [µM]	MS-HRM					
		M1/U1			M3/U3		
		% DNA methylation	Fold change	*p*-value*	% DNA methylation	Fold change	*p*-value*
C	0.1	4.92	0.428	0.0074	18.28	0.851	0.8725
	1	5.72	0.498	0.0255	17.43	0.811	0.6282
	10	3.78	0.330	0.0012	17.11	0.797	0.5298
	100	5.68	0.495	0.0241	17.94	0.836	0.7866
EC	0.1	3.06	0.267	0.0003	15.33	0.714	0.1453
	1	4.81	0.419	0.0062	17.37	0.809	0.6125
	10	2.71	0.236	0.0002	14.71	0.685	0.0827
	100	1.52	0.133	0.0002	12.94	0.602	0.0346
EGC	0.1	10.83	0.943	0.9995	23.91	1.113	0.9841
	1	6.28	0.547	0.0560	19.71	0.918	0.9959
	10	20.30	1.768	0.0002	28.58	1.331	0.0593
	100	7.84	0.682	0.3482	22.65	1.055	0.9993
ECG	0.1	9.90	0.862	0.9896	12.80	0.596	0.0111
	1	5.49	0.478	0.0181	17.53	0.816	0.6616
	10	21.69	1.889	<0.0001	30.28	1.410	0.0095
	100	18.33	1.596	0.0047	26.42	1.230	0.3679
EGCG	0.1	7.39	0.643	0.3428	19.06	0.888	0.9849
	1	6.53	0.569	0.0787	18.95	0.882	0.9766
	10	3.09	0.269	0.0016	19.77	0.920	0.9989
	100	11.52	1.003	>0.9999	14.50	0.675	0.0677
GSH	0.1	6.16	1.067	0.9976	15.43	0.942	0.9292
	1	6.92	1.199	0.8934	19.06	1.163	0.3171
	10	5.83	1.009	>0.9999	19.51	1.191	0.2087
	100	3.88	0.672	0.6301	16.44	1.003	>0.9999

* statistical analysis was performed using one-way ANOVA with Dunnett's test with a cut at $p < 0.05$

Table S5. Changes in SRXN1 gene expression analysed with RT-qPCR method. Expression levels were calculated using relative delta delta Ct method. Data was normalized to GAPDH gene expression. The results are means ± SD of three biological replicates.

Sample	Average relative mRNA levels	SD	*p*-value*	Fold change	Fold regulation	Fold regulation from profiler [9]
Control	0.0218	0.0090	-	0	0	0
EGC 10	0.0052	0.0060	0.0294	0.238	-4.209	-2.542
ECG 10	0.0186	0.0063	0.8662	0.855	-1.170	-1.592
ECG 100	0.0102	0.0008	0.1243	0.467	-2.141	-

* statistical analysis was performed using one-way ANOVA with Dunnett's test with a cut at $p < 0.05$

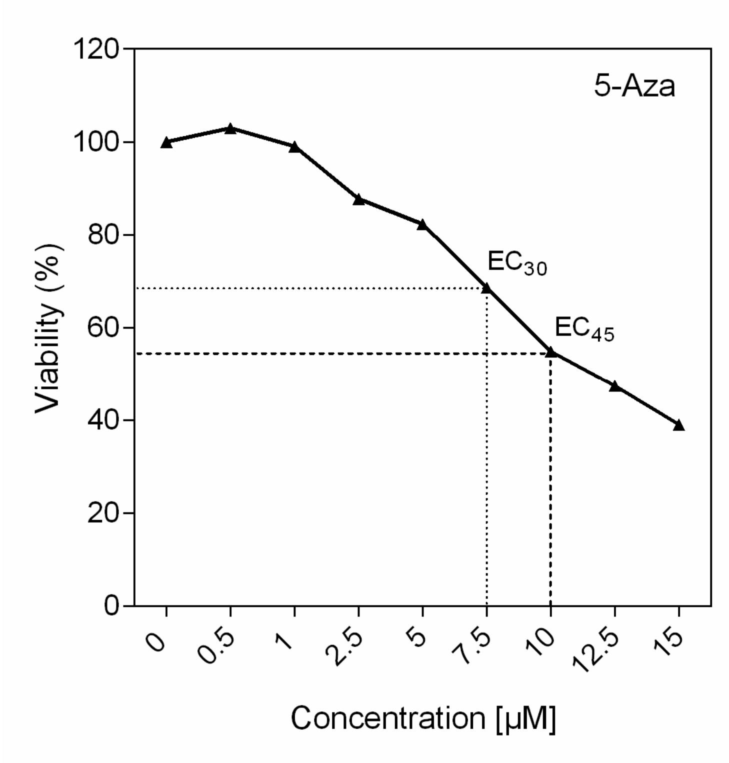

Figure S1. Inhibition of growth of HT29 cells determined by MTT assay after 72 h treatment with 5-Aza. Viability is expressed as a percentage relative to control cells (100%). EC_{30} and EC_{45} are the effective concentrations of 30% and 45% of cell growth inhibition. Results are means of three independent experiments carried out in triplicate (SD < 15%).

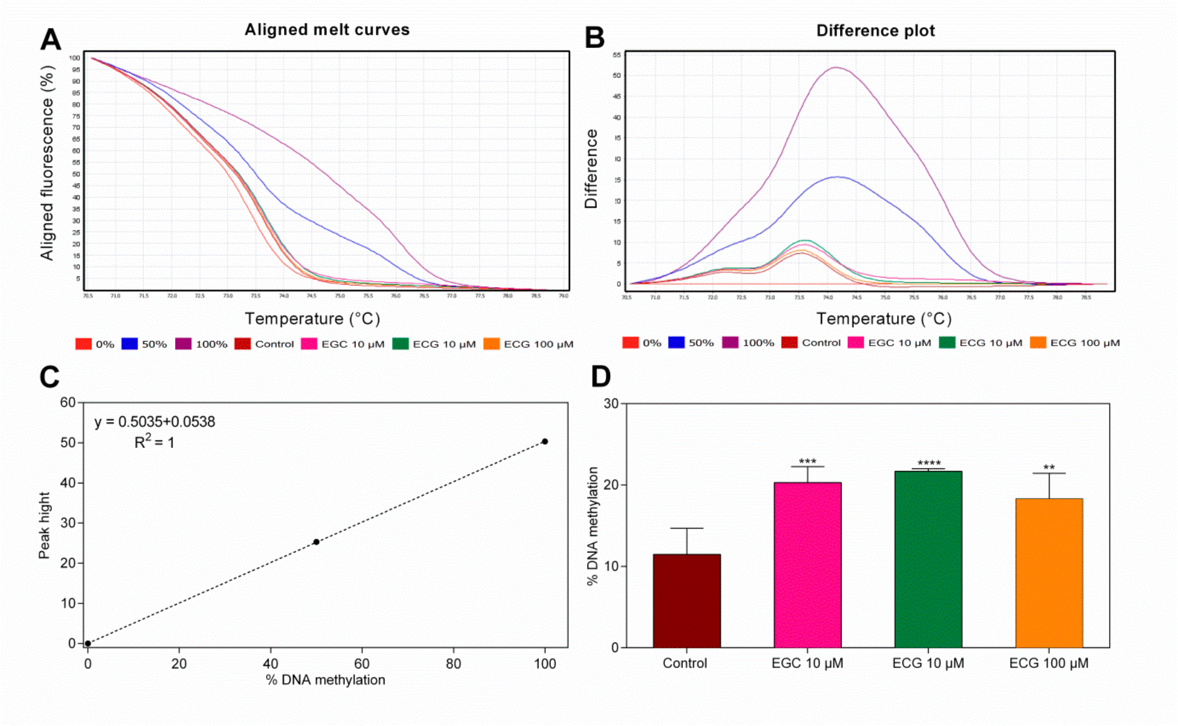

Figure S2. The increase in DNA methylation level of SRXN1 gene determined by MS-HRM using M1/U1 primer sets following 24 h treatment of HT29 cells with catechins that increase methylation profile of SRXN1 promoter. (A) The printouts document the representative aligned melt curves and (B) difference plots showing positions of control, EGC 10 μM, ECG 10 μM and ECG 100 μM with respect to 0, 50 and 100% methylated standards. (C) Standard curve and linear regression equation were obtained by plotting the peak heights of standards (extracted from difference curves aligned against unmethylated control (0%)) and percentage of DNA methylation. (D) Bar graph presenting the increase in DNA methylation levels based on standard curves for EGC 10 μM, ECG 10 μM and ECG 100 μM. The asterisks mark *p*-values as follows: (**)—≤ 0.01, (***)—≤ 0.001, and (****)—≤ 0.0001.

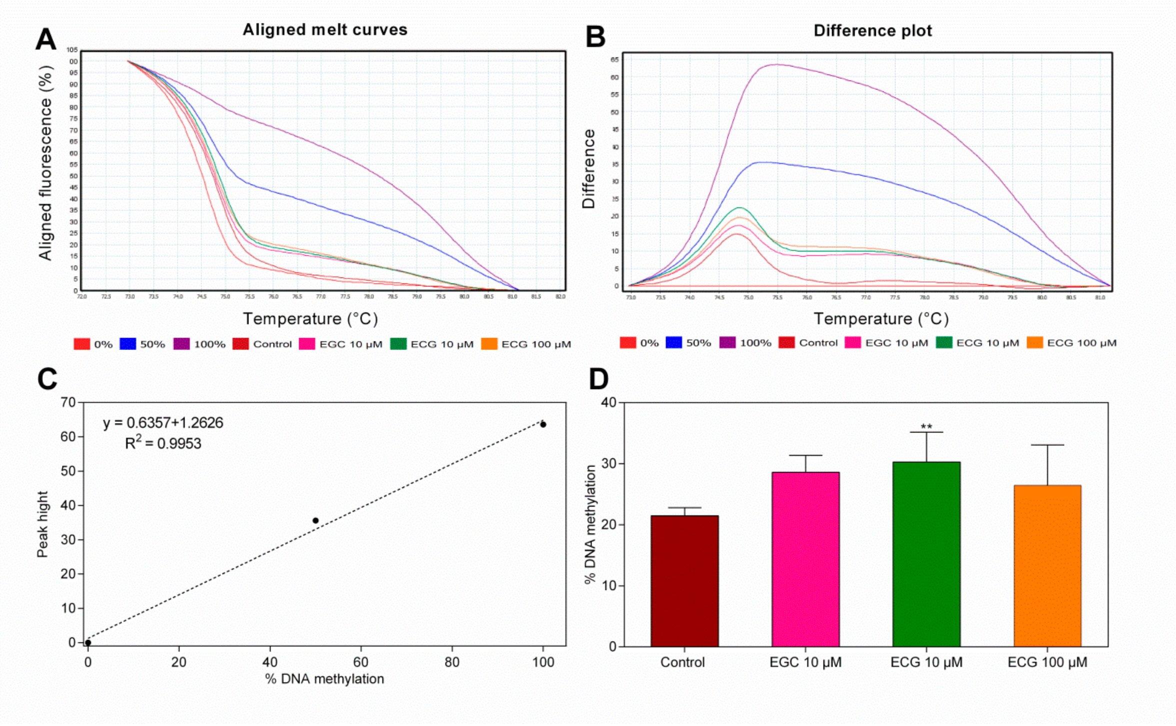

Figure S3. The increase in DNA methylation level of SRXN1 gene determined by MS-HRM using M3/U3 primer sets following 24 h treatment of HT29 cells with catechins that increase methylation profile of SRXN1 promoter. (A) The printouts document the representative aligned melt curves and (B) difference plots showing positions of control, EGC 10 μM, ECG 10 μM and ECG 100 μM with respect to 0, 50 and 100% methylated standards. (C) Standard curve and linear regression equation were obtained by plotting the peak heights of standards (extracted from difference curves aligned against unmethylated control (0%)) and percentage of DNA methylation. (D) Bar graph presenting the increase in DNA methylation levels based on standard curves for EGC 10 μM, ECG 10 μM and ECG 100 μM. The asterisks mark *p*-values as follows: (**)—≤ 0.01.
